# Tetrapyrrole Signaling in Plants

**DOI:** 10.3389/fpls.2016.01586

**Published:** 2016-10-19

**Authors:** Robert M. Larkin

**Affiliations:** Key Laboratory of Horticultural Plant Biology (Ministry of Education), College of Horticulture and Forestry Sciences, Huazhong Agricultural UniversityWuhan, China

**Keywords:** tetrapyrrole, porphyrin, heme, Mg-protoporphyrin IX, plastid signaling, plastid, chloroplast, chlorophyll

## Abstract

Tetrapyrroles make critical contributions to a number of important processes in diverse organisms. In plants, tetrapyrroles are essential for light signaling, the detoxification of reactive oxygen species, the assimilation of nitrate and sulfate, respiration, photosynthesis, and programed cell death. The misregulation of tetrapyrrole metabolism can produce toxic reactive oxygen species. Thus, it is not surprising that tetrapyrrole metabolism is strictly regulated and that tetrapyrrole metabolism affects signaling mechanisms that regulate gene expression. In plants and algae, tetrapyrroles are synthesized in plastids and were some of the first plastid signals demonstrated to regulate nuclear gene expression. In plants, the mechanism of tetrapyrrole-dependent plastid-to-nucleus signaling remains poorly understood. Additionally, some of experiments that tested ideas for possible signaling mechanisms appeared to produce conflicting data. In some instances, these conflicts are potentially explained by different experimental conditions. Although the biological function of tetrapyrrole signaling is poorly understood, there is compelling evidence that this signaling is significant. Specifically, this signaling appears to affect the accumulation of starch and may promote abiotic stress tolerance. Tetrapyrrole-dependent plastid-to-nucleus signaling interacts with a distinct plastid-to-nucleus signaling mechanism that depends on GENOMES UNCUOPLED1 (GUN1). GUN1 contributes to a variety of processes, such as chloroplast biogenesis, the circadian rhythm, abiotic stress tolerance, and development. Thus, the contribution of tetrapyrrole signaling to plant function is potentially broader than we currently appreciate. In this review, I discuss these aspects of tetrapyrrole signaling.

## Introduction

**FIGURE 1:**
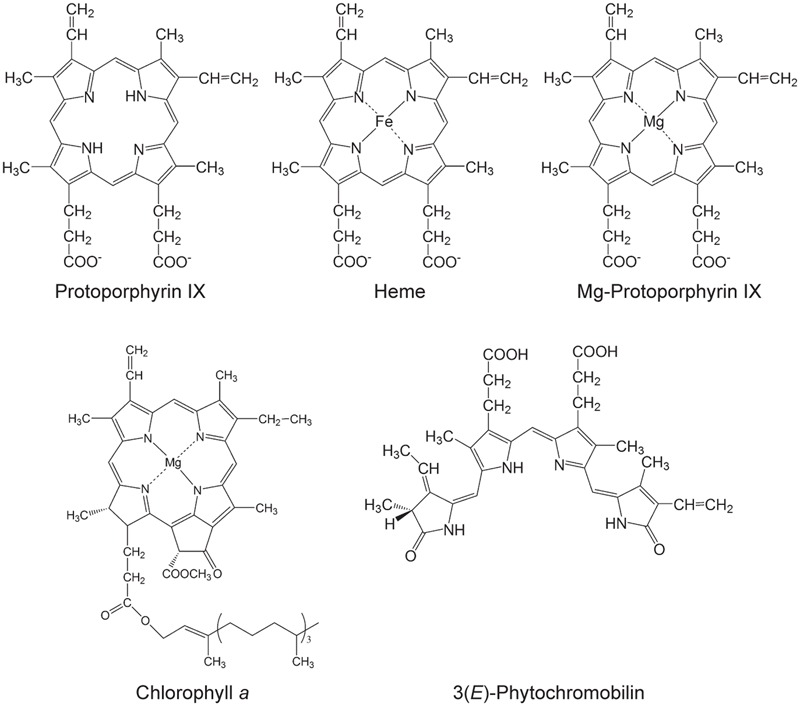
**Selected tetrapyrroles from plants.** The structures were adapted from Tanaka and Tanaka (2007).

Tetrapyrroles contribute a number of functions to processes that are critical for the viability of diverse organisms including essential functions to the light reactions photosynthesis, which underpins life on earth. Tetrapyrroles contain four pyrroles— aromatic rings containing four carbon atoms and one nitrogen atom—often linked together with methine groups, in linear (e.g., bilins) or cyclic arrangements (e.g., porphyrins). Porphyrins chelate metal ions, such as cobalt, iron, or magnesium ions. These structural features along with a variety of ring substituents contribute to the diverse functions of tetrapyrroles (Tanaka and Tanaka, 2007; Tanaka et al., 2011; Brzezowski et al., 2015; **Figure 1**).

**FIGURE 2:**
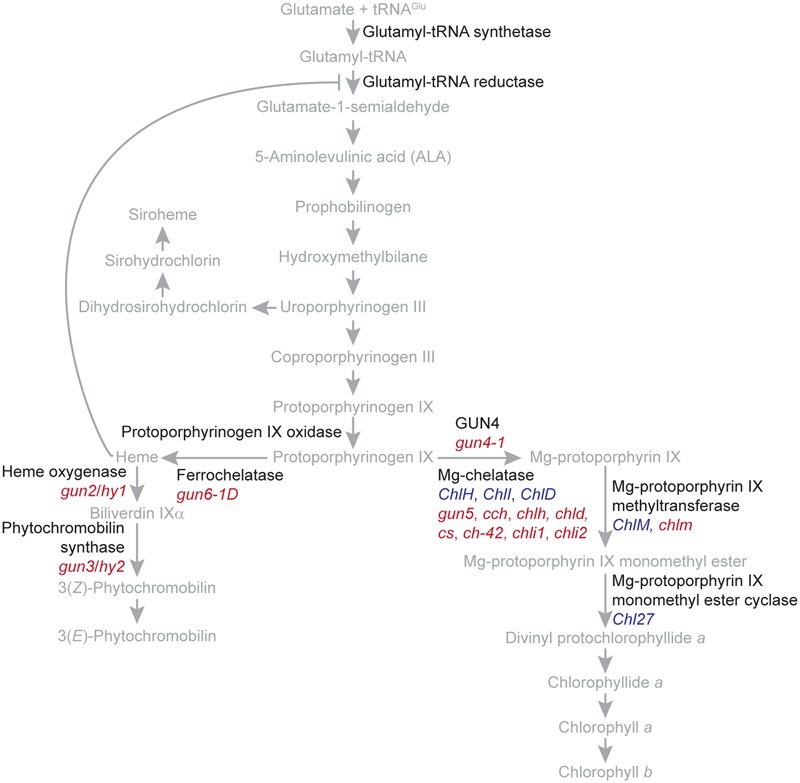
**The branched tetrapyrrole biosynthetic pathway of plants.** The names of the precursors and end products of the branched tetrapyrrole biosynthetic pathway are indicated with gray. Enzyme catalyzed reactions are indicated with gray arrows. The feedback inhibition of glutamyl tRNA reductase by heme is indicated with a gray T bar. The names of proteins, such as regulatory proteins and enzymes, are indicated with black. The tetrapyrrole biosynthetic pathway was adapted from Tanaka and Tanaka (2007) and Tanaka et al. (2011). Genes encoding enzymes or enzyme subunits are indicated with blue. Mutants discussed in the text are indicated with red. *gun5, cch*, and *chlh* are mutant alleles of *ChlH*/*GUN5*. *chld* is a mutant allele of *ChlD*. *cs, ch-42*, and *chli1* are mutant alleles of *ChlI1. chli2* is a mutant allele of *ChlI2*.

In plants and algae, tetrapyrroles are synthesized ultimately from glutamate by a branched biosynthetic pathway localized entirely within plastids. The end products of this branched pathway are siroheme, heme, phytochromobilin, chlorophyll *a*, and chlorophyll *b* (**Figure 2**). These tetrapyrroles provide critical functions to important processes, such as light signaling, the detoxification of reactive oxygen species, the assimilation of nitrate and sulfate, respiration, programmed cell death, and the light-harvesting reactions of photosynthesis. *In vivo*, many tetrapyrroles, such as porphyrins, are sequestered to prevent them from generating singlet oxygen (^1^O_2_), a toxic reactive oxygen species that causes stress and cell death. Electronically excited versions of porphyrins produce ^1^O_2_ when they transfer energy to molecular oxygen (Tanaka and Tanaka, 2007; Tanaka et al., 2011; Brzezowski et al., 2015).

Considering the large number of critical functions performed by tetrapyrroles and the potentially lethal consequences of misregulating tetrapyrrole biosynthesis, it is not surprising that the levels of tetrapyrroles are strictly controlled (Tanaka and Tanaka, 2007; Tanaka et al., 2011; Brzezowski et al., 2015) and that tetrapyrroles affect signaling mechanisms that regulate gene expression in diverse organisms (Terry and Smith, 2013; Brzezowski et al., 2015; Zhang et al., 2015). Indeed, tetrapyrroles were some of the first molecules suggested to serve as plastid signals that regulate nuclear gene expression in both plants and algae (Susek and Chory, 1992). The idea that in addition to serving as the metabolic centers of plant cells, plastids serve as sensors that emit signals that regulate gene expression in the nucleus was first suggested almost forty years ago (Bradbeer et al., 1979). Since the early days of plastid-to-nucleus signaling research, researchers have studied the plastid regulation of photosynthesis-associated nuclear genes (PhANGs), especially the genes encoding the type I proteins of the major light harvesting complex of photosystem II (Lhcb1) (Susek and Chory, 1992; Gray et al., 2003). The Lhcb1 proteins accumulate in the thylakoid membranes where they bind chlorophylls, carotenoids, and serve as antennae for photosystem II (PSII) (Jansson et al., 1992). The expression of *Lhcb* genes is well studied. Additionally, when chloroplasts experience dysfunction, the expression of *Lhcb* genes is severely downregulated relative to other PhANGs (Susek and Chory, 1992).

Since the early days of plastid-to-nucleus signaling research, mutant alleles, and inhibitors that specifically block chloroplast biogenesis by a variety of distinct mechanisms were used to induce the plastid-to-nucleus signaling mechanisms that downregulate the expression of *Lhcb* genes and other PhANGs (Susek and Chory, 1992; Gray et al., 2003). Chloroplast biogenesis refers to the development of chloroplasts from non-photosynthetic proplastids. Chloroplast biogenesis occurs during germination and leaf development (Pogson et al., 2015). Subsequently, chloroplast division and a mechanism that establishes the size of the chloroplast compartment drive the proliferation of chloroplasts (Osteryoung and Pyke, 2014; Larkin et al., 2016). Experiments with inhibitors and mutant alleles that block chloroplast biogenesis indicated that the expression of PhANGs depends on chloroplast biogenesis and that the plastid-to-nucleus signaling that is activated by blocking chloroplast biogenesis typically downregulates the transcription of PhANGs (Susek and Chory, 1992; Gray et al., 2003). However, there is evidence for plastid signals using a posttranscriptional mechanism to regulate the expression of a PhANG. The plastid regulated expression of *PetE* from pea requires an mRNA with an intact 5′ end and depends on translation in transgenic tobacco and transgenic *Arabidopsis* (Sullivan and Gray, 2002; Brown et al., 2005). During the early days of plastid-to-nucleus signaling research, a porphyrin was considered an attractive candidate for a plastid signal that regulates nuclear gene expression because at the time, heme was known to regulate nuclear gene expression in *Saccharomyces cerevisiae* (Forsburg and Guarente, 1989) and an inverse correlation was reported between the levels of the chlorophyll precursor Mg-protoporphyrin IX monomethyl ester (Mg-ProtoME) and the levels of the mRNAs that encode the Lhcb proteins and the small subunit of RuBisCO in *Chlamydomonas reinhardtii* (Johanningmeier and Howell, 1984).

Now, there is a larger body of evidence favoring the idea that porphyrins and bilins serve as plastid signals that regulate nuclear gene expression in both plants and algae (Terry and Smith, 2013; Brzezowski et al., 2015; Zhang et al., 2015). Additionally, we now know that there are a number of non-porphyrin plastid signals that activate distinct plastid-to-nucleus signaling mechanisms. We also know that plastid-to-nucleus signaling is not only a feedback mechanism that optimizes chloroplast function but that plastid-to-nucleus signaling affects extraplastidic processes (Larkin, 2014; Chan et al., 2016). In addition to affecting entire cells by regulating nuclear gene expression, plastid signals affect individual chloroplasts by promoting the degradation of individual chloroplasts that suffer from photooxidative stress, apparently by stimulating the ubiquitination of chloroplast proteins that are located on the cytosolic surface of chloroplasts (Woodson et al., 2015). Indeed, plastid-to-nucleus signaling mechanisms broadly contribute to plant function by serving as major regulators of plant cells.

Plastid-to-nucleus signaling research is frequently and extensively reviewed. Here, I review the data indicating that porphyrins regulate nuclear gene expression and the evidence for particular mechanisms of porphyrin-regulated gene expression in plants. I also review possible biological functions for porphyrin-dependent plastid-to-nucleus signaling and our understanding of the interactions between a porphyrin-dependent signaling mechanism with a signaling mechanism that does not appear to require porphyrins for its activation.

## Early Experiments Relevant to Tetrapyrrole Signaling in Plants

During the early days of plastid-to-nucleus signaling research, a number experiments were performed that tested whether porphyrins might contribute to the plastid regulation of nuclear gene expression in plants, but there were major technical obstacles. First, plants were thought to not take up porphyrins, such as the chlorophyll precursor Mg-ProtoME that was implicated as a plastid signal in *C. reinhardtii* (Johanningmeier and Howell, 1984), or similar porphyrins, such as Mg-protoporphyrin IX (Mg-Proto), and hemin. Second, the porphyrin precursors of chlorophyll, such as Mg-Proto and Mg-ProtoME, are potent photosensitizers. Third, the model genes for studying plastid-to-nucleus signaling were PhANGs. The expression of PhANGs is strongly induced by qualities and quantities of light that would induce severe photooxidative stress if porphyrins were to accumulate and diffuse freely throughout plant cells.

Researchers overcame these technical barriers by feeding 5-aminolevulinic acid (ALA) to plants. ALA is the 5-carbon universal precursor of tetrapyrroles. ALA is hydrophilic and is readily taken up by plants. Exogenous ALA was demonstrated to flood the tetrapyrrole biosynthetic pathway with intermediates, induce increases in the levels of chlorophyll precursors (Granick, 1959; Gough, 1972; Mascia, 1978), and downregulate the expression of *Lhcb* genes in *Lepidium sativum* and *Arabidopsis thaliana* seedlings in non-photosensitizing conditions, such as after a non-photosensitizing pulse of red light, continuous illumination with non-photosensitizing far-red light, or the dark (Kittsteiner et al., 1991; Vinti et al., 2000). These data provide evidence that the accumulation of chlorophyll precursors, such as Mg-Proto or Mg-ProtoME, or that the accumulation of other plastid-derived tetrapyrroles regulate the expression of PhANGs in plants. Similar results were obtained after treating plants with inhibitors that induce increases in the levels of Mg-ProtoMe and Mg-Proto in non-photosensitizing conditions (Oster et al., 1996; La Rocca et al., 2001). More recently, ALA was suggested to mediate the effects of Mg-porphyrin metabolism on nuclear gene expression (Czarnecki et al., 2012)

## The *gun* Mutant Screen

Developing mutant screens that specifically interrogate particular mechanisms is an objective approach for identifying completely unknown mechanisms. Thus, developing a specific mutant screen was an attractive approach for studying the plastid-to-nucleus signaling that is activated when chloroplast biogenesis is blocked. Developing specific mutant screens remains an attractive approach for studying diverse plastid-to-nucleus signaling mechanisms because our current knowledge of plastid-to-nucleus signaling mechanisms indicates that many plastid-to-nucleus signaling mechanisms are not intuitively obvious (Chan et al., 2016). The first specific mutant screen developed that specifically interrogates plastid-to-nucleus signaling mechanisms was the *genomes uncoupled* (*gun*) mutant screen (Susek and Chory, 1992; Susek et al., 1993). *gun* mutant screens use reporter genes to screen for *Arabidopsis* mutants that transcribe elevated levels of PhANGs, such as *Lhcb* genes, when chloroplast biogenesis is blocked with an herbicide treatment (Susek et al., 1993; Koussevitzky et al., 2007; Ruckle et al., 2007; Cottage et al., 2008; Woodson et al., 2011). *gun* alleles do not affect the expression of PhANGs in green seedlings containing well-functioning chloroplasts. *gun* alleles may attenuate the activity of negative regulators of PhANG expression, or *gun* alleles may induce the activity of positive regulators of PhANG expression (Susek et al., 1993; Mochizuki et al., 2001; Koussevitzky et al., 2007; Ruckle et al., 2007; Woodson et al., 2011). In addition to regulating the expression of PhANGs when chloroplast biogenesis is blocked, the plastid-to-nucleus signaling that depends on the *GUN* genes was shown to regulate the expression of PhANGs when chloroplast and etioplast function is somewhat attenuated (Koussevitzky et al., 2007; Sun et al., 2011; Woodson et al., 2013; Tadini et al., 2016).

*gun* mutant screens yielded a large number of mutant alleles of genes that encode a chloroplastic pentatricopeptide repeat protein named GUN1 (Susek et al., 1993; Koussevitzky et al., 2007; Cottage et al., 2008), a photoreceptor that is activated by blue light named cryptochrome1 (cry1) (Ruckle et al., 2007), and genes that contribute to tetrapyrrole metabolism (Mochizuki et al., 2001; Larkin et al., 2003; Adhikari et al., 2011). These data indicate that *gun* mutant screens specifically disrupt a few distinct mechanisms that downregulate the expression of *Lhcb* genes when chloroplast biogenesis is blocked and that when chloroplast biogenesis is blocked, specific mechanisms downregulate the expression of PhANGs. The specificity of the *gun* mutant screen was first demonstrated more than 20 years ago (Susek et al., 1993). The data from Susek et al. (1993) and the data from subsequent analyses of *gun* mutant screens (Mochizuki et al., 2001; Larkin et al., 2003; Koussevitzky et al., 2007; Ruckle et al., 2007; Cottage et al., 2008; Adhikari et al., 2011) conflict with a commonly promoted alternative interpretation that unnatural and complex metabolism downregulates the expression of PhANGs when chloroplast biogenesis is blocked (Pfannschmidt, 2010; Voigt et al., 2010; Barajas-López et al., 2012; Kindgren et al., 2012; Terry and Smith, 2013).

## *Gun2, Gun3, Gun4, Gun5*, and *Gun6*

The *gun* screen yielded several mutant alleles of genes that contribute to tetrapyrrole metabolism named *GUN2, GUN3, GUN4, GUN5*, and *GUN6* (**Figure 2**). *GUN2* encodes heme oxygenase. *GUN3* encodes phytochromobilin synthase (Mochizuki et al., 2001). These enzymes contribute to the biosynthesis of phytochromobilin, the chromophore of phytochromes (Tanaka and Tanaka, 2007). *gun2* and *gun3* are allelic to *hy1* and *hy2*, which disrupt photomorphogenesis (Koornneef et al., 1980). A *hy1* mutant was independently demonstrated to exhibit a *gun* phenotype (Vinti et al., 2000). *GUN5* encodes the 140-kDa subunit of Mg-chelatase (Mochizuki et al., 2001). *GUN4* encodes an activator of Mg-chelatase that binds the 140-kDa subunit of Mg-chelatase and the porphyrin substrate and product of Mg-chelatase (Larkin et al., 2003; Davison et al., 2005; Verdecia et al., 2005; Adhikari et al., 2009). GUN4 is only found in organisms that perform oxygenic photosynthesis (Larkin et al., 2003). GUN4 was demonstrated to shield porphyrins from collisions with molecular oxygen that produce ^1^O_2_ (Brzezowski et al., 2014), as was suggested previously (Larkin et al., 2003; Verdecia et al., 2005). However, the subsequent finding that porphyrin-binding pocket of GUN4 is partially opened (Chen et al., 2015) and that *in vitro*, porphyrins produce more ^1^O_2_ when they are bound to GUN4 (Tarahi Tabrizi et al., 2016) indicates that GUN4 requires additional factors to shield porphyrins from collisions with molecular oxygen. Thus, GUN4 and GUN5 divert protoporphyrin IX from heme biosynthesis to chlorophyll biosynthesis by inserting a magnesium ion into protoporphyrin IX, yielding Mg-Proto. *GUN6* encodes ferrochelatase1 (FC1), which synthesizes heme from protoporphyrin IX (Woodson et al., 2011). The cloning of so many mutant alleles that disrupt tetrapyrrole metabolism indicates that *gun* mutant screens specifically disrupt a plastid-to-nucleus signaling mechanism that is regulated by tetrapyrrole metabolism. Consistent with these data, porphyrins serve as plastid signals in diverse organisms. Null alleles of *GUN4* affected nuclear gene expression in *C. reinhardtii*—even in dark-grown cultures (Formighieri et al., 2012), both Mg-Proto and heme were reported to serve as plastid signals in algae (Beck, 2005; von Gromoff et al., 2008; Voß et al., 2011; Tanaka and Hanaoka, 2013), and heme was demonstrated to regulate various signaling mechanisms in diverse organisms, such as bacteria, red algae, yeast, and animals (Tanaka and Hanaoka, 2013; Terry and Smith, 2013; Kobayashi et al., 2016).

In the *gun* mutant screen, norflurazon is typically used to block chloroplast biogenesis. This herbicide specifically inhibits phytoene desaturase, which plays an essential role in carotenoid biosynthesis by catalyzing two sequential dehydrogenation reactions on phytoene, yielding ζ-carotene. Thus, norflurazon treatments inhibit the accumulation of carotenoids. Carotenoids are essential for quenching triplet chlorophyll and ^1^O_2_ in green tissues (Susek and Chory, 1992; Gray et al., 2003; Nisar et al., 2015). Thus, when green leaves are treated with norflurazon, ^1^O_2_ accumulates and leaves suffer from severe photooxidative stress (Kim and Apel, 2013). However, in the *gun* mutant screen, *Arabidopsis* seeds are germinated on a growth medium containing norflurazon and subsequently, seedlings are grown for several days on the same medium until their gene expression phenotypes are scored. In these conditions, chlorophyll does not accumulate, the thylakoid membranes do not develop, chloroplast biogenesis appears arrested at an early stage, and plastids resemble proplastids (Susek et al., 1993). Norflurazon was proposed to block chloroplast biogenesis by causing photooxidative stress during the conversion of proplastids to chloroplasts (Susek and Chory, 1992; Gray et al., 2003). This model assumes that when seedlings are germinated on a medium containing norflurazon—during the biogenesis of chloroplasts from non-photosynthetic proplastids—free chlorophyll or free chlorophyll precursors accumulate without the concomitant accumulation of carotenoids. This model assumes that these chlorophylls or chlorophyll precursors drive the accumulation of ^1^O_2_ and that the resulting photooxidative stress blocks chloroplast biogenesis (Susek and Chory, 1992; Gray et al., 2003).

A variety of independent experiments—including experiments with ^1^O_2_-detecting dyes and ^1^O_2_-regulated genes—unambiguously indicated that ^1^O_2_ accumulates in green *Arabidopsis* leaves treated with norflurazon (Kim and Apel, 2013). In stark contrast, the same methods for detecting ^1^O_2_ indicated that ^1^O_2_ does not appear to accumulate when *Arabidopsis* seeds are germinated on a medium containing norflurazon (Kim and Apel, 2013). One interpretation of these data is that norflurazon might use a different mechanism to photobleach green leaves and to block chloroplast biogenesis during germination. For instance, the coordinated downregulation of both carotenoid and chlorophyll biosynthesis was suggested to block chloroplast biogenesis by preventing the biogenesis of the thylakoid membranes, without inducing photooxidative stress (Kim and Apel, 2013). Indeed, neither chlorophyll nor its precursors accumulate when *Arabidopsis* seedlings are germinated on a medium containing norflurazon (Mochizuki et al., 2008; Moulin et al., 2008).

An alternative interpretation is that a transient production of ROS blocks chloroplast biogenesis when *Arabidopsis* seeds are germinated on a medium containing norflurazon and because of the transient nature of this increase in the levels of ROS, these ROS are impractical or impossible to detect (Mochizuki et al., 2010; Kim and Apel, 2013). Consistent with this interpretation, when seeds were germinated on a medium containing norflurazon, the expression of one ^1^O_2_-inducible gene was higher after 3 days than after 5 days of growth (Kim and Apel, 2013). However, changes in the expression of more than one ^1^O_2_-inducible gene are required to provide evidence for elevated levels of ^1^O_2_ because genes are typically regulated by more than one signal. Nonetheless, light influences the ability of norflurazon to downregulate the expression of nuclear genes that are regulated by plastid signals, which provides more evidence for a mechanism that depends on photooxidative stress (Susek and Chory, 1992). For this reason, norflurazon-treated seedlings are typically grown in bright white light (e.g., at least 100 μmol m^-2^ s^-1^) to test for *gun* phenotypes. However, norflurazon appeared to block chloroplast biogenesis when *Arabidopsis* seedlings were grown in 10 μmol m^-2^ s^-1^ white light (Voigt et al., 2010). The impact of chloroplast function on light signaling (Lepistö and Rintamäki, 2012; Larkin, 2014) may at least partially explain the effect of the fluence rate on norflurazon treatments. Light signaling generally induces the expression of PhANGs in green seedlings but when chloroplast biogenesis is blocked, light signaling becomes a negative regulator of *Lhcb* expression (Ruckle et al., 2007 and Figure 1 from Ruckle et al., 2012) and severely attenuates the expression of many other PhANGs (Ruckle et al., 2012). This conversion of light signaling from a positive to a negative regulator of *Lhcb* genes while only attenuating the light-induced expression of other PhANGs may explain why blocking chloroplast biogenesis with norflurazon treatments downregulates the expression of *Lhcb* genes more than other PhANGs (Susek and Chory, 1992).

A number of authors have promoted the idea that the *gun* mutants with deficiencies in tetrapyrrole metabolism are partially resistant to norflurazon without discussing the data that conflict with this interpretation. Their interpretation is that norflurazon is less effective in mutants that are partially chlorophyll deficient because lower levels of chlorophyll biosynthesis reduce the amount of ROS produced during chloroplast biogenesis in norflurazon-treated seedlings. Therefore, in these *gun* mutants, they propose that the norflurazon-induced inhibition of chloroplast biogenesis is not as severe as in wild-type seedlings. Consequentially, they propose that PhANGs are expressed at higher levels in these *gun* mutants than in wild type because PhANG expression is correlated with chloroplast biogenesis (Mochizuki et al., 2008; Moulin et al., 2008; Kleine et al., 2009; Galvez-Valdivieso and Mullineaux, 2010; Mochizuki et al., 2010; Armbruster et al., 2011; Jarvis and López-Juez, 2013; Kleine and Leister, 2013; Terry and Smith, 2013; Bobik and Burch-Smith, 2015).

The first indication that the chlorophyll-deficient *gun* mutants are not resistant to norflurazon was the lack of correlation between chlorophyll-deficient phenotypes and the *gun* phenotypes, which was apparent after the characterization of a number of tetrapyrrole biosynthesis mutants from *Arabidopsis*. For instance, the 40-kDa subunit of Mg-chelatase is encoded by two genes named *ChlI1* and *ChlI2*. A *ChlI1* mutant named *ch42-1* was reported to accumulate 11% of the chlorophyll found in wild type or to not accumulate chlorophyll (Mochizuki et al., 2001; Rissler et al., 2002). When *ChlI2* was knocked out, chlorophyll levels were significantly less but similar to wild type (Kobayashi et al., 2008). Thus, *CHLI1* encodes the predominant isoform of this Mg-chelatase subunit. *cs* is a leaky loss-of-function allele of *ChlI1* (Koncz et al., 1990). *cs* and *gun4-1* accumulated less than 40% of the chlorophyll found in wild type. *cch* and *gun5* are *GUN5* mutants that accumulated approximately 35 and 70% of the chlorophyll found in wild type (Mochizuki et al., 2001). In contrast to *cch, gun4-1*, and *gun5*, neither *cs* nor *ch42-1* expressed higher levels of *Lhcb1* than wild type when chloroplast biogenesis was blocked with a norflurazon treatment (i.e., *cch, gun4-1*, and *gun5* are *gun* mutants. *cs* and *ch42-1* are not *gun* mutants) (Mochizuki et al., 2001). Additionally, the *gun* phenotype appeared significantly attenuated in the completely albino *hy1 gun5* double mutant relative to the partially chlorophyll deficient *gun5* and *hy1* (i.e., *gun2*) mutants (Vinti et al., 2000). Thus, there is no correlation between chlorophyll deficient phenotypes and *gun* phenotypes.

T-DNA insertion alleles of the genes encoding the subunits of Mg-chelatase that induce severe chlorophyll deficiencies or albinism also induce *gun* phenotypes (Huang and Li, 2009). In contrast to previous work that used northern blots to test for *gun* phenotypes (Vinti et al., 2000; Mochizuki et al., 2001), Huang and Li (2009) quantified the expression of *Lhcb1* genes using qRT-PCR. They found an approximately twofold increase in *Lhcb1* expression in the severely chlorophyll-deficient *chli1* mutants and 4.5- to 8-fold increases in the albino *chld* and *chlh* mutants, which are null alleles of the genes encoding the 80- and 140-kDa subunits of Mg-chelatase, respectively. The finding that *CHLI1* mutants were either not *gun* mutants or were subtle *gun* mutants depending on whether *Lhcb1* expression was quantified by northern blotting or by qRT-PCR and that *CHLH* mutants exhibited robust *gun* phenotypes regardless of the method used for quantifying *Lhcb1* expression are essentially the same results.

Additional data conflict with the idea that partial resistance to norflurazon explains the *gun* phenotypes of the *gun* mutants that have defects in tetrapyrrole metabolism. (1) Attempts to show that norflurazon-treated *gun4-1* and *gun5* accumulate less reactive oxygen species (ROS) than norflurazon-treated wild type by quantifying ROS-responsive gene expression, staining with ROS responsive dyes, and by treating plants with ROS scavengers were not successful (Strand et al., 2003; Voigt et al., 2010; Zhang et al., 2011a; Kim and Apel, 2013). (2) No *gun* mutants were obtained from screens for norflurazon resistant mutants (i.e., the *happy on norflurazon* mutants) (Saini et al., 2011). (3) *gun4-1* and *gun5* were not resistant to low concentrations of norflurazon, in contrast to the *happy on norflurazon* mutants (Saini et al., 2011). (4) Blocking chloroplast biogenesis with a norflurazon treatment activated plastid-to-nucleus signaling mechanisms that are distinct from the ^1^O_2_-dependent plastid-to-nucleus signaling mechanisms that were activated when green seedlings were treated with norflurazon and consequentially the levels of chloroplastic ^1^O_2_ were increased (Kim and Apel, 2013). (5) High-fluence-rate light and a ROS-inducing methyl viologen treatment required different promoter elements than norflurazon treatments that blocked chloroplast biogenesis to downregulate the expression of the *Lhcb1^∗^2* promoter from *Nicotiana plumbaginifolia* (Staneloni et al., 2008). (6) Null alleles of *sigma factor2* (*sig2*) reduced chlorophyll levels to 20% of the chlorophyll found in wild type. Thus, in contrast to norflurazon treatments, *sig2* attenuates but does not block chloroplast biogenesis. *sig2* mutants expressed lower levels of PhANGs than wild type. Etiolated *gun5 sig2* expressed higher levels *Lhcb2.2* and other PhANGs than etiolated *sig2* (Woodson et al., 2013). Thus, perturbing tetrapyrrole metabolism affected nuclear gene expression in dark-grown *Arabidopsis* seedlings that were not treated with norflurazon. (7) Tetrapyrrole metabolism regulated starch-related nuclear gene expression in Bright Yellow-2 (BY2) cells derived from tobacco, which lack photosynthetic plastids (Enami et al., 2011). (8) In *C. reinhardtii*, mutant alleles of *GUN4* and *ChlM*—the gene encoding Mg-protoporphyrin IX methyltransferase—upregulated the expression of genes that encode the LHC proteins and genes that contribute to tetrapyrrole metabolism in cells that were not treated with norflurazon (Meinecke et al., 2010; Formighieri et al., 2012; Brzezowski et al., 2014). (9) Phytoene desaturase was inhibited in norflurazon-treated *gun* mutants with deficiencies in tetrapyrrole metabolism, as a class. Thus, alternative mechanisms of partial resistance do not explain the phenotypes of these *gun* mutants (Voigt et al., 2010).

The idea that no evidence of partial resistance to norflurazon was obtained in so many independent experiments because during chloroplast biogenesis, the norflurazon-induced increase in ROS is transient or localized (Mochizuki et al., 2010; Kim and Apel, 2013) is difficult to reconcile with the data. This interpretation is especially difficult to reconcile with the findings that there was no correlation between the chlorophyll-deficient phenotypes and the *gun* phenotypes (Vinti et al., 2000; Mochizuki et al., 2001), etiolated *gun5 sig2* expressed higher levels of several PhANGs than etiolated *sig2* (Woodson et al., 2013), and neither *gun4-1* nor *gun5* were resistant to low concentrations of norflurazon—in contrast to norflurazon-resistant mutants (Saini et al., 2011). If norflurazon treatments block chloroplast biogenesis in *Arabidopsis* seedlings by inducing photooxidative stress, (1) chloroplast biogenesis in *Arabidopsis* seedlings would need to exhibit such extreme sensitivity to this photooxidative stress that the perturbations in the tetrapyrrole biosynthetic pathway exhibited by *gun2, gun3, gun4-1, gun5*, and *gun6-1D* would not affect the photobleaching activity of norflurazon and (2) the perturbations in tetrapyrrole metabolism induced by *gun5* would need to affect a distinct mechanism to affect the expression of PhANGs in the dark.

## The Mechanism of Tetrapyrrole Signaling in Plants: Chlorophyll Biosynthetic Enzymes and Chlorophyll Precursors

The mutant alleles of *GUN2*/*HY1, GUN3*/*HY2, GUN4*, and *GUN5* that were isolated from *gun* mutant screens are loss-of-function alleles (Mochizuki et al., 2001; Larkin et al., 2003; Adhikari et al., 2011). In contrast, the mutant allele of *GUN6* that was isolated by Woodson et al. (2011) is a gain-of-function allele. These mutant alleles reduce the flux through the chlorophyll branch by two distinct mechanisms: (1) directly inhibiting chlorophyll biosynthesis or (2) promoting the accumulation of heme. Heme inhibits chlorophyll biosynthesis by attenuating the activity of glutamyl tRNA reductase. These mutant alleles may also induce increases in the levels of heme by diverting protoporphyrin IX from the chlorophyll branch to the heme branch, increasing ferrochelatase activity, or by attenuating the activities of heme oxygenase and phytochromobilin synthase (Vinti et al., 2000; Mochizuki et al., 2001; Larkin et al., 2003; Woodson et al., 2011).

Based on the finding that chlorophyll biosynthesis is attenuated in *gun4, gun5, cch, cs*, and *ch42-1*, and that in contrast to *gun4, gun5*, and *cch*, neither *cs* nor the *ch42-1* are *gun* mutants, tetrapyrrole-dependent plastid-to-nucleus signaling was proposed to not depend on the accumulation of a particular chlorophyll precursor, such as Mg-Proto or Mg-ProtoME. Instead, GUN5 was proposed to participate in plastid-to-nucleus signaling (Mochizuki et al., 2001). The turnover of a porphyrin, such as Mg-Proto, or changes in the subchloroplastic localization of GUN4, GUN5, or a GUN4-GUN5 complex associated or not associated with a porphyrin ligand was suggested affect plastid-to-nucleus signaling (Mochizuki et al., 2001, 2008, 2010; Larkin et al., 2003; Sun et al., 2011). Consistent with these ideas, a number of proteins perform more than one function, such as distinct functions in metabolism and signaling (Boukouris et al., 2016). Indeed, the ortholog of GUN5 from *Synechocystis* sp. PCC 6803 serves as both a Mg-chelatase subunit and as an anti-sigma factor (Osanai et al., 2009).

The finding that porphyrin binding increases the affinity of GUN4 and GUN5 for chloroplast membranes (Adhikari et al., 2009, 2011) is also consistent with these ideas because the chloroplast envelope membrane may serve as an important subchloroplastic location for communication between chloroplasts and extrachloroplastic compartments.

Subsequently, the levels of Mg-Proto were reported to accumulate when chloroplast biogenesis was blocked with a norflurazon treatment, and Mg-Proto was reported to accumulate to lower levels in *gun2* and *gun5* (Strand et al., 2003). Data from a number of additional experiments performed by Strand et al. (2003) supported the idea that the accumulation of Mg-Proto is a plastid signal that downregulates PhANG expression, including an experiment showing that exogenous Mg-Proto downregulated the expression of *Lhcb* genes in protoplasts and that exogenous hemin did not affect the expression of *Lhcb* genes in protoplasts. The data from Strand et al. (2003) conflict with the data reported by Mochizuki et al. (2001) and provide evidence that the chlorophyll-deficient *gun* mutants affect plastid-to-nucleus signaling by reducing the levels of Mg-Proto. Subsequently, other work provided more evidence that the accumulation of a particular Mg-porphyrin might serve as a plastid signal. A barley mutant deficient in the Mg-ProtoME cyclase accumulated Mg-ProtoME and did not exhibit a *gun* phenotype (Gadjieva et al., 2005). An *Arabidopsis* mutant deficient in the Mg-ProtoME cyclase accumulated Mg-Proto and did not exhibit a *gun* phenotype (Ankele et al., 2007). An *Arabidopsis* mutant deficient in Mg-Proto methyltransferase accumulated Mg-Proto and did not exhibit a *gun* phenotype (Pontier et al., 2007). Based on these data, these authors suggested that the accumulation of either Mg-Proto (Ankele et al., 2007; Pontier et al., 2007) or Mg-ProtoME (Gadjieva et al., 2005) might serve as a plastid signal that downregulates the expression of PhANGs. Modulating the levels of Mg-Proto methyltransferase with sense or antisense expression in tobacco was reported to affect PhANG expression, which is also consistent with Mg-porphyrins serving as a plastid signal (Alawady and Grimm, 2005). Using confocal laser scanning spectroscopy, Mg-Proto or Mg-ProtoME were detected in the cytosol of ALA fed *Arabidopsis* plants that were treated or not treated with norflurazon (Ankele et al., 2007; Zhang et al., 2011a). In one instance, Mg-Proto or Mg-ProtoME were detected in the cytosol of cells from plants that were not fed with ALA (Zhang et al., 2011a). Thus, plastids are capable of exporting Mg-Proto or Mg-ProtoME. These data are consistent with plastids exporting Mg-Proto or Mg-ProtoME and with Mg-Proto or Mg-ProtoME activating a signaling mechanism in the cytosol that regulates the expression of PhANGs.

As discussed above, researchers commonly elevated the endogenous levels of porphyrins with exogenous ALA because plant roots were thought to not take up porphyrins, such as Mg-Proto. More recently, *Arabidopsis* plants were reported to take up Mg-Proto through their roots when they were simply watered with solutions containing Mg-Proto (Zhang et al., 2011a; Kindgren et al., 2012; Barajas-López et al., 2013). An increase in the efficiency of Mg-Proto and hemin feeding was reported by first vacuum infiltrating seedlings prior to root feeding (Zhang et al., 2013). In one instance, feeding Mg-Proto to plants through their roots was reported to induce an increase in the areal levels of Mg-Proto (Kindgren et al., 2012). Exogenous Mg-Proto, Mg-ProtoME, or hemin doubled the cellular RNA content in *Arabidopsis* plants (Zhang et al., 2011b). Feeding both Mg-Proto and hemin to plants downregulated the expression of *Lhcb* and *RbcS* genes (Zhang et al., 2011a, 2013; Kindgren et al., 2012; Barajas-López et al., 2013). This reduced expression of PhANGs that was induced by exogenous porphyrins was attenuated in *gun1, long hypocotyl5* (*hy5*), *aba-insentive4* (*abi4*), and *phytochrome-associated protein phosphatase 5* (*papp5*) (Zhang et al., 2011a; Kindgren et al., 2012; Barajas-López et al., 2013). These findings are consistent with tetrapyrrole metabolism affecting plastid-to-nucleus signaling upstream of the chloroplastic pentatricopeptide protein GUN1 and with PAPP5 and the nuclear transcription factors HY5 and ABI4 contributing to this mechanism. Exogenous porphyrins affecting GUN1-dependent plastid-to-nucleus signaling is consistent with purified chloroplasts importing porphyrins *in vitro* (Adhikari et al., 2009, 2011). Feeding Mg-Proto and hemin to plants was also reported to affect the expression of genes encoding transcription factors, such as CAAT binding proteins (CBP) and the C-REPEAT BINDING FACTORS (CBF) (Zhang et al., 2013; Norén et al., 2016). In apparent conflict with these data collected from intact *Arabidopsis* plants, Strand et al. (2003) reported that exogenous hemin did not affect the expression of *Lhcb* in *Arabidopsis* protoplasts.

In stark contrast, three different laboratories demonstrated that Mg-Proto did not accumulate in barley and *Arabidopsis* when chloroplast biogenesis was blocked with norflurazon treatments (Gadjieva et al., 2005; Mochizuki et al., 2008; Moulin et al., 2008). Additionally, the norflurazon-treated *chlm gun4* and the *chlm gun5* double mutants accumulated elevated levels of Mg-Proto relative to *gun4, gun5*, and wild type but exhibited the same *gun* phenotypes as *gun4* and *gun5* when chloroplast biogenesis was blocked with a norflurazon treatment (Mochizuki et al., 2008). Exogenous ALA induced an approximately 200-fold increase in the levels of Mg-Proto and Mg-ProtoME in etiolated *Arabidopsis* seedlings but had no impact on the subsequent far-red light-induced expression of *Lhcb1* (Mochizuki et al., 2008). Elevated levels of Mg-Proto in transgenic rice plants that overexpress *Myxococcus xanthus* protoporphyrinogen IX oxidase did not reduced the levels of *Lhcb* expression relative to wild type (Phung et al., 2011). Moulin et al. (2008) suggested that the difference in the levels of Mg-Proto in norflurazon-treated seedlings reported by Strand et al. (2003) and Moulin et al. (2008) resulted from Strand et al. (2003) misidentifying a contaminating substance as Mg-Proto. Alternatively, a transient accumulation of Mg-Proto was suggested to explain this discrepancy (Kindgren et al., 2011, 2012). Indeed, transient accumulations of Mg-Proto were reported when plants were grown in photoperiodic light (Pöpperl et al., 1998; Papenbrock et al., 1999; Norén et al., 2016) or when chloroplasts experience photooxidative stress (Aarti et al., 2006; Kindgren et al., 2011, 2012; Zhang et al., 2011a). The problem with the proposal that a transient accumulation of Mg-Proto explains this discrepancy is that the growth conditions used to detect the *gun* phenotype do not fluctuate (i.e., constant temperature and continuous light of a constant fluence rate) to eliminate the possibility of the circadian regulation of PhANG expression obscuring the plastid regulation of PhANG expression (Susek et al., 1993). Indeed, transient peaks of Mg-Proto were not observed in plants grown in continuous light (Pöpperl et al., 1998; Papenbrock et al., 1999; Norén et al., 2016).

An alternative to the idea that a sustained accumulation of Mg-Proto in the chloroplast regulates gene expression in the nucleus is that a transient increase in the levels of Mg-Proto might regulate gene expression in the nucleus. Indeed, transient changes in the levels of Mg-Proto and hemin affect plastid-to-nucleus signaling in algae (Kropat et al., 2000; von Gromoff et al., 2008; Kobayashi et al., 2009; Voß et al., 2011). In 3-week-old green *Arabidopsis* plants, a transient increase in the levels of Mg-Proto and Mg-ProtoME occurred 48 h after an oxidative-stress inducing norflurazon treatment (Zhang et al., 2011a). Similarly, oxidative stress resulting from a methyl viologen treatment induced a rise in the levels of Mg-Proto and Mg-ProtoME in cucumber and *Arabidopsis* (Aarti et al., 2006; Kindgren et al., 2011, 2012). Both treatments appeared to inhibit the activity of the Mg-ProtoME cyclase (Aarti et al., 2006; Zhang et al., 2011a; Kindgren et al., 2011, 2012). These herbicide treatments downregulated the expression of *Lhcb* genes (Zhang et al., 2011a; Kindgren et al., 2011, 2012). Zhang et al. (2011a) provided evidence that hydrogen peroxide and superoxide might not downregulate the expression of *Lhcb* genes by acting downstream of GUN5. In contrast, Kindgren et al. (2011, 2012) did not account for potential ROS effects on the expression of *Lhcb* genes. Zhang et al. (2011a) concluded that norflurazon treatments induce a transient increase in the levels of Mg-Proto in 3-week-old plants and based on their data in 3-week-old plants inferred that in the *gun* mutant screen, norflurazon might induce a transient increase in the levels of Mg-Proto that leads to the long-term down-regulation of *Lhcb* expression. Kim and Apel (2013) performed an experiment with 5-day-old *Arabidopsis* seedlings that was similar to the experiment performed by Zhang et al. (2011a) with 3-week-old plants. Five-day-old seedlings were treated with norflurazon for 12 h in the dark and then returned to continuous light. This treatment induced increases in the levels of superoxide anion radical, hydrogen peroxide, ^1^O_2_, and ^1^O_2_-dependent plastid-to-nucleus signaling (Kim and Apel, 2013) but had no effect on the expression of *Lhcb* genes 24 h after the norflurazon-treated seedlings were transferred from the dark to the light. The different ages or the different methods of treating *Arabidopsis* plants with norflurazon may explain why Zhang et al. (2011a) observed a decrease in *Lhcb* expression 24 h after transferring norflurazon treated plants to bright light and Kim and Apel (2013) did not.

In contrast, Schlicke et al. (2014) induced a transient increase in Mg-porphyrin levels without first inducing photooxidative stress. They downregulated the expression of the genes encoding the 140-kDa subunit of Mg-chelatase (*ChlH*), Mg-Proto methyltransferase (*ChlM*), and Mg-ProtoME cyclase (*Chl27*) in 10-day-old *Arabidopsis* seedlings with an inducible RNAi system. Within 24 h, Mg-ProtoME accumulated—to higher levels than observed by Zhang et al. (2011a)—in the *Chl27* RNAi lines. It is not clear whether the approach of Zhang et al. (2011a) or Schlicke et al. (2014) induced larger increases in the levels of Mg-Proto. In the *Chl27* RNAi lines—24 h after suppression was induced—there were no macroscopic changes in the plants and no significant differences in the expression of PhANGs or ROS-regulated genes. Four days of downregulated *Chl27*expression induced chlorophyll deficiencies, necrosis, and downregulated PhANG expression. Plastid-to-nucleus signaling was proposed to not cause the changes in gene expression that were observed 24 h after the RNAi-induced suppression of *ChlH, ChlM*, and *Chl27* (Schlicke et al., 2014).

When plants are grown in photoperiodic light, transient increases in the levels of Mg-Proto and Mg-ProtoME routinely occur—apparently without causing photooxidative stress. For example, at dawn in tobacco and barley, Mg-Proto accumulated 30 (barely) and 60-fold (tobacco). Mg-ProtoME accumulated 100 (tobacco) and 200-fold (barley). This accumulation occurred within 30 min (tobacco) and 60 min (barley) and declined within another hour (Pöpperl et al., 1998). An independent study of the accumulation of Mg-Proto and Mg-ProtoME in tobacco in photoperiodic light appeared to miss the first striking peaks of Mg-Proto and Mg-ProtoME detected by Pöpperl et al. (1998) but appeared to detect the subsequent broad peak of Mg-Proto and Mg-ProtoME accumulation that occurred throughout the remainder of the light period (Papenbrock et al., 1999) that was also reported by Pöpperl et al. (1998). A similar broad peak of Mg-Proto and Mg-ProtoME accumulation was reported in *Arabidopsis* grown in photoperiodic light (Norén et al., 2016). In tobacco, increases in the expression of *Lhcb* genes preceded dawn, only increased for 6 h, and began declining 8 h after dawn (Paulsen and Bogorad, 1988), several hours after the transient and striking increases in the levels of Mg-Proto and Mg-ProtoME (Pöpperl et al., 1998). Thus, in tobacco, there is no correlation between the diurnal accumulation of these Mg-porphyrins (Pöpperl et al., 1998; Papenbrock et al., 1999) and the levels of *Lhcb* mRNA (Paulsen and Bogorad, 1988).

An attempt to identify the cytosolic receptor for Mg-Proto in plants revealed that a large number of different cytosolic proteins bound to an affinity column constructed by linking Mg-Proto to Affi-Gel 102 (Kindgren et al., 2011). Several different types of heat shock proteins bound to this column (Kindgren et al., 2011). The affinities of these heat shock proteins for Mg-Proto were not quantified. Although the finding that the HEAT SHOCK PROTEIN 90 (HSP90) bound to a Mg-Proto-affinity column and the finding that 20 μM Mg-Proto induced a 16% reduction in the ATPase activity of HSP90 provide evidence that HSP90s bind Mg-Proto (Kindgren et al., 2011, 2012), these data do not provide evidence for the high-affinity binding expected for a receptor.

Based on precedents for heat shock proteins contributing to heme signaling in yeast and a previously demonstrated connection between HY5 and both plastid-to-nucleus signaling and *Lhcb* expression, Kindgren et al. (2012) provided evidence that both HSP90 and HY5 contribute to the Mg-Proto-regulated expression of *Lhcb* genes. RNAi lines that reduced the expression of all *HSP90.1*–*HSP90.4* genes exhibited lower levels of PhANG expression relative to wild type, but only norflurazon- or methyl viologen-treated lines were tested (Kindgren et al., 2012). Although *gun* mutants exhibit defects in PhANG expression when chloroplasts experience dysfunction, *gun* mutants do not exhibit defects in PhANG expression when plants contain well-functioning chloroplasts. Thus, whether these effects of RNAi suppression of HSP90 are caused by specific defects in plastid-to-nucleus signaling or by broad effects on the transcriptome would appear important to test. Indeed, HSP90s broadly contribute to the transcriptome, development, and environmental responses (Sangster et al., 2007). Additionally, the idea that HY5 specifically contributes to Mg-Proto signaling conflicts with the finding that HY5 was a *gun* mutant when chloroplast biogenesis was blocked with a lincomycin treatment (Ruckle et al., 2007). Lincomycin treatments do not appear to activate the plastid-to-nucleus signaling that depends on tetrapyrroles (Gray et al., 2003; Koussevitzky et al., 2007). Subsequently, phytochrome-associated protein phosphatase 5 (PAPP5)—previously identified as a regulator of phytochrome signaling (Ryu et al., 2005) and as a protein that associates with a Mg-Proto Affi-Gel 102 column (Kindgren et al., 2011)—was demonstrated to serve as a negative regulator of chloroplast function by a variety of assays in green plants not treated with norflurazon. Consistent with this finding *Lhcb 2.4* and other PhANGs were expressed at higher levels in *papp5* than in wild type. A connection to Mg-Proto signaling was suggested because *papp5* specifically increased the levels of chlorophyll in a chlorophyll-deficient Mg-ProtoME cyclase mutant and because the downregulation of *Lhcb2.4* expression by exogenous Mg-Proto and exogenous ALA was attenuated in *papp5* relative to wild type (Barajas-López et al., 2013). Thus, if Mg-Proto is a plastid signal, potential components of the signaling mechanism are not unique to Mg-Proto signaling. With the addition of ZEITLUPE and PSEUDO-RESPONSE REGULATOR5, this HSP90 and HY5-dependent mechanism was proposed to help plastid signals converge with the circadian clock and regulate the expression of *C-CBF* genes in *Arabidopsis* (Norén et al., 2016).

Distinct mechanistic information on porphyrin signaling was reported by Zhang et al. (2013). They provided evidence that interactions between porphyrins and GUN1 promote the binding of ABI4 to the promoters of genes that encode Lhcb and CCAAT binding factor A (CBFA). ABI4 was reported to inhibit the transcription of these genes by competitively binding to a *cis*-element that a distinct transcription activator binds when ABI4 is absent. Additionally, reduced levels of CBFA promoted the accumulation of the LEAFY COTYLEDON1 subunit of the heme activator protein complex (HAP), which binds to promoters of genes encoding HAP and stimulates their transcription. HAP and CBP induced transcription in general and thus, were proposed to promote the transient increases in the levels of cellular RNA that porphyrin signaling is proposed to induce (Zhang et al., 2013). Although a role for ABI4 acting downstream of GUN1 was repeatedly reported (Koussevitzky et al., 2007; Sun et al., 2011; Zhang et al., 2013; Xu et al., 2016), other work provides evidence that additional nuclear transcription factors contribute to the plastid-to-nucleus signaling that is regulated by the *GUN* genes, such as HY5 and GLK1 (Acevedo-Hernández et al., 2005; Ruckle et al., 2007; Kakizaki et al., 2009; Waters et al., 2009; Kerchev et al., 2011).

## The Mechanism of Tetrapyrrole Signaling in Plants: Heme

An alternative to the idea that Mg-Proto serves as plastid signal is that heme serves as a plastid signal. For example, the accumulation and export of heme from the chloroplast to the cytosol or nucleus, or changes in the chloroplastic levels or subchloroplastic location of a particular pool of heme might serve as a plastid signal that induces the expression of PhANGs. In this model, the inductive heme signal is emitted from well-functioning chloroplasts and plastids do not emit this inductive signal when chloroplast biogenesis is blocked or attenuated. The main evidence that heme serves as plastid signal is (1) that overexpressing catalytically active FC1 within the physiologically relevant range and feeding ALA to norflurazon-treated seedlings induced an increase in the expression of PhANGs (Woodson et al., 2011) and (2) that *gun5*, overexpressing *FC1*, and exogenous ALA induced the expression of PhANGs in *sig2* mutants that were not treated with norflurazon (Woodson et al., 2013). *SIG2* is required for the synthesis of tRNA^Glu^ in plastids (Schön et al., 1986; Kanamaru et al., 2001). The synthesis of glutamyl tRNA from glutamate and tRNA^Glu^ is the first step in the tetrapyrrole biosynthesis (Tanaka and Tanaka, 2007; Tanaka et al., 2011). The levels tRNA^Glu^ and glutamyl tRNA were each reduced in *sig2*, which links *SIG2* to tetrapyrrole biosynthesis and signaling (Woodson et al., 2013). Consistent with heme serving as an inductive plastid signal, the expression of ferrochelatase from *Bradyrhizobium japonicum* in the cytosol of rice was suggested to induce increases in the ALA synthesizing capacity of chloroplasts by activating a signaling mechanism that regulates gene expression in the nucleus (Kim et al., 2014).

There are two isoforms of ferrochelatase in *Arabidopsis*. The expression of *FC1* is observed in non-photosynthetic tissues and is induced by stress. *FC2* is coexpressed with PhANGs (Tanaka et al., 2011). Although plants that overexpressed *FC1* exhibited *gun* phenotypes, plants that overexpressed *FC2* did not exhibit *gun* phenotypes (Woodson et al., 2011). These data provide more evidence that plastid-to-nucleus signaling is activated by changes in the location or concentration of a particular pool of a particular porphyrin—perhaps a transient change in concentration—(Mochizuki et al., 2008, 2010; Woodson et al., 2011) and that this porphyrin is heme (Woodson et al., 2011, 2013).

Consistent with heme serving as a plastid signal in plants, chloroplasts are known to synthesize and export heme to a variety of subcellular locations (Thomas and Weinstein, 1990; Cornah et al., 2003; Tanaka et al., 2011), and heme serves as a signal that regulates gene expression in diverse organisms (Tanaka and Hanaoka, 2013; Terry and Smith, 2013; Kobayashi et al., 2016). Nonetheless, there are data that appear to conflict with the idea that porphyrin-dependent plastid-to-nucleus signaling is explained by an increase in the level of heme serving as a plastid signal. For instance, although elevated levels of heme were reported in *gun2* relative to wild type and in *gun5 sig2* relative to *sig2*, heme was reported to not accumulate above wild-type levels in *gun4, gun5*, or in plants that overexpress *FC1* (Woodson et al., 2011, 2013; Espinas et al., 2012). There are conflicting data on whether heme accumulates in norflurazon-treated *gun2* relative to norflurazon-treated wild type and whether heme accumulates in untreated *Arabidopsis* seedlings relative to norflurazon-treated seedlings (Voigt et al., 2010; Woodson et al., 2011). The caveat with these conflicting data is that different methods for quantifying the levels of free heme (i.e., heme that is not associated with proteins and that may accumulate in cellular membranes) can yield different results. Indeed, an analysis of methods for extracting and quantifying heme from plants indicated that the level of free heme is not correlated with the *gun* phenotype. Thus, heme-dependent plastid-to-nucleus signaling does not appear to depend on the accumulation of free heme. The heme that activates plastid-to-nucleus signaling was proposed to bind to a protein that contributes to a signaling mechanism (Espinas et al., 2012).

Although the data favoring a simple heme-based signaling mechanism is compelling, ALA feeding experiments provide evidence that heme signaling is complex or that porphyrin signaling is more complex than a heme signaling mechanism. Although exogenous ALA induced increases in the expression of PhANGs in norflurazon-treated seedlings (Woodson et al., 2011) and although feeding low quantities of ALA to etiolated *sig2* and *sig6* seedlings induced increases in expression of PhANGs, feeding high quantities of ALA to etiolated *sig2* and *sig6* seedlings downregulated the expression of *Lhcb*. Additionally, feeding low quantities of ALA to etiolated wild-type seedlings had no inductive effect on *Lhcb* expression and feeding larger quantities of ALA to etiolated wild-type seedlings downregulated the expression of *Lhcb* (Woodson et al., 2013). Exogenous ALA either upregulated or downregulated the expression of PhANG expression depending on the age of *Lepidium sativum* seedlings grown in far-red light (Kittsteiner et al., 1991). Feeding ALA to both wild-type and *hy1* seedlings downregulated the expression of *Lhcb* in the dark and in far-red light (Vinti et al., 2000; Czarnecki et al., 2012). Feeding hemin to intact plants either downregulated or had no effect on the expression of *Lhcb* (Zhang et al., 2011a, 2013). Some of the apparently contradictory results from ALA feeding experiments are potentially explained by light-regulated development affecting porphyrin signaling (Woodson et al., 2013). Alternatively, Zhang et al. (2013) suggested that the repressive effect of high concentrations of exogenous ALA and heme on PhANG expression and that the inductive effect of low concentrations of exogenous ALA and the overexpression of *FC1* on PhANG expression provides evidence that heme can either downregulate or upregulate the expression of PhANGs depending on whether heme is present at high or low levels in the cell. Although some reports of exogenous ALA downregulating the expression of PhANGs is potentially explained by non-specific and complex effects of unnaturally high levels of porphyrins on PhANG expression, extremely high porphyrin levels would appear necessary to explain a non-specific mechanism or toxicity that is not based on ROS because 200-fold increases in the levels of Mg-Proto and Mg-ProtoME that were induced by exogenous ALA were reported to not affect the expression of *Lhcb1* (Mochizuki et al., 2008).

In the conditions used for the *gun* mutant screen, the effects of the *gun4* and *gun5* alleles on nuclear gene expression are potentially explained by the influence of the defects in Mg-porphyrin biosynthesis on heme biosynthesis (Papenbrock et al., 2000; Alawady and Grimm, 2005; Woodson et al., 2011). Conversely, the data supporting the idea that heme serves as an inductive plastid signal in seedlings containing well-functioning chloroplasts (Woodson et al., 2011, 2013) is also consistent with increased heme biosynthesis affecting the chlorophyll branch of the tetrapyrrole biosynthetic pathway. Perhaps increases in heme biosynthesis might affect the subchloroplastic localization of GUN4, GUN5, or a GUN4-GUN5 complex bound to or not bound to a porphyrin ligand, which might serve as a repressive signal (Woodson et al., 2011).

## Interactions Between Tetrapyrrole Signaling and Gun1-Dependent Signaling

The plastid-to-nucleus signaling mechanism that is affected by tetrapyrrole metabolism appears to interact with the plastid-to-nucleus signaling that depends on GUN1. Tetrapyrrole-dependent and GUN1-dependent plastid-to-nucleus signaling mechanisms appeared to downregulate the expression of PhANGs in a partially redundant manner because a synergistic enhancement of the *gun* phenotype was observed in the *gun1-1 gun4-1* and the *gun1-1 gun5* double mutants relative to the single mutants (Mochizuki et al., 2001). However, the alleles used by Mochizuki et al. (2001) were leaky. Nonetheless, there was no evidence that the *gun* phenotype was enhanced in the *gun4-1 gun5* double mutant relative to the single mutants (Mochizuki et al., 2001). Subsequently, a double mutant was constructed with *gun5* and *gun1-9*, a non-sense allele of *GUN1*. The *gun* phenotype of this double mutant was indistinguishable from *gun1-9*. These data provide evidence that the tetrapyrrole signal acts upstream of GUN1 (Koussevitzky et al., 2007). The synergistic increase in the *gun* phenotype observed by Mochizuki et al. (2001) was suggested to result from leaky alleles (Koussevitzky et al., 2007). Transcriptome analyses were also consistent with significant interactions between the plastid signaling mechanisms disrupted by mutant alleles of *GUN1* and *GUN5* (Koussevitzky et al., 2007). The finding that exogenous Mg-Proto downregulated the expression of *RbcS* and *Lhcb* in wild type but not in *gun1, papp5, abi4*, or *hy5* (Zhang et al., 2011a; Kindgren et al., 2012; Barajas-López et al., 2013) provides more evidence that tetrapyrrole-dependent plastid signaling acts upstream of GUN1. In contrast, exogenous hemin downregulated the expression of a gene that encodes the CBP in *gun1-9* in 3-week-old *Arabidopsis* plants (Zhang et al., 2013). Thus, heme signaling may not absolutely require GUN1 in 3-week-old *Arabidopsis* plants.

The finding that there were increases in the levels of ALA, heme, and chlorophyll in *gun1 sig2* relative to *sig2* provides more evidence for interactions between these two plastid-to-nucleus signaling mechanisms (Woodson et al., 2013). Consistent with these and other data, GUN1 was suggested to act upstream of porphyrin biosynthesis and to affect plastid-to-nucleus signaling by serving as a negative regulator of tetrapyrrole biosynthesis (Terry and Smith, 2013). In this model for plastid-to-nucleus signaling, GUN1 acts upstream of tetrapyrrole biosynthesis rather than downstream of tetrapyrrole biosynthesis as previously suggested (Koussevitzky et al., 2007). The finding that although *gun1* and *gun4-1* suppress the chlorophyll-deficient phenotype of *slow green1, gun5* enhances the chlorophyll-deficient phenotype of *slow green1* (Hu et al., 2014) provides evidence for a complex interaction between tetrapyrrole metabolism and GUN1. The finding that light-regulated development affects porphyrin-dependent plastid-to-nucleus signaling (Woodson et al., 2013) indicates additional complexity in this type of plastid-to-nucleus signaling.

In general, pentatricopeptide repeat proteins, such as GUN1, contribute to the expression of the plastidic and mitochondrial genomes (Barkan and Small, 2014). The simplest interpretation of the current data is that the influence of GUN1 on plastid-to-nucleus signaling is probably related to a role for GUN1 in the expression of the chloroplast genome and that GUN1 and tetrapyrrole metabolism interact by an indirect mechanism because the expression of the chloroplast genome and tetrapyrrole metabolism are distinct processes. However, direct interactions between GUN1 and tetrapyrrole metabolism seem possible because the biochemical function of GUN1 is poorly understood and because there is considerable genetic evidence for interactions between GUN1 and tetrapyrrole metabolism. Heme was suggested to possibly interact with GUN1-dependent plastid-to-nucleus signaling by affecting PHD type transcription factor with transmembrane domains (PTM), which acts downstream of GUN1 (Sun et al., 2011; Terry and Smith, 2013).

One approach for learning more about the biochemical function of GUN1 and the interactions between GUN1 and tetrapyrrole metabolism is to identify other molecules that bind GUN1, such as other proteins. However, purifying a multisubunit protein complex containing native GUN1 is potentially difficult because GUN1 is thought to accumulate at low levels (Liu et al., 2013). Tadini et al. (2016) provided evidence that GUN1 and the enzymes that perform tetrapyrrole biosynthesis might directly interact by performing two hybrid and BiFC experiments and by purifying a GFP-tagged version of GUN1 from plants that overexpressed GUN1-GFP after treating chloroplasts that were purified from these plants with a crosslinking agent. GUN1 is capable of interacting with four proteins that contribute to tetrapyrrole biosynthesis—the 79-kDa subunit of Mg-chelatase (also known as ChlD), porphobilinogen deaminase, uroporphyrinogen III decarboxylase, and ferrochelatase I—when these proteins are expressed at abnormally high levels in two-hybrid and BiFC experiments. Nearly 300 different proteins that contribute to diverse chloroplastic processes were crosslinked to GUN1-GFP after GUN1-GFP was overexpressed in *Arabidopsis*. One of these crosslinked proteins was ChlD. Thus, GUN1 has a propensity for interacting with many other proteins when it is overexpressed in yeast and plants (Tadini et al., 2016). The relevance of these numerous interactions for native GUN1 expressed at normal levels in wild-type plants is not known.

## Biological Function of Tetrapyrrole Signaling in Plants

The finding that in addition to *Arabidopsis*, both maize and barley mutants that are deficient in Mg-chelatase exhibited *gun* phenotypes (Burgess and Taylor, 1988; Gadjieva et al., 2005) indicates that selection pressure has maintained this signaling mechanism in diverse plants and that the influence of tetrapyrrole metabolism on plastid-to-nucleus signaling serves an important function in plants. Nonetheless, the biological function of tetrapyrrole-dependent plastid-to-nucleus signaling is not well established.

Although the influence of tetrapyrrole-dependent plastid-to-nucleus signaling on the expression of PhANGs is commonly studied, transcriptome analyses of *C. reinhardtii* are consistent with tetrapyrrole-dependent plastid-to-nucleus signaling not serving as a major regulator of PhANG expression. Indeed, the largest group of genes regulated by Mg-Proto and hemin in *C. reinhardtii* contribute to proteolysis and protein folding. The expression of more than half of these genes is regulated by heat shock. Based on these data, porphyrin signaling was proposed to help *C. reinhardtii* adapt to changing environmental conditions (Voß et al., 2011). The finding that the overexpression of stress-inducible *FC1* but not the overexpression of photosynthesis-associated *FC2* affects plastid-to-nucleus signaling in *Arabidopsis* is consistent with porphyrin-dependent plastid-to-nucleus signaling affecting non-photosynthetic and stress-related functions in *Arabidopsis* (Woodson et al., 2011). Nonetheless, transcriptome analyses provided evidence that porphyrin-dependent plastid-to-nucleus signaling affects PhANG expression in *Arabidopsis* (Strand et al., 2003). Unfortunately, a plant system similar to *C. reinhardtii* in the sense that tetrapyrrole-dependent plastid-to-nucleus signaling is rapidly induced without introducing complicating secondary effects, such as ROS, does not exist.

Tetrapyrrole-dependent plastid-to-nucleus signaling may broadly affect plant cells. Heme signaling appears to affect the expression of genes that contribute to starch biosynthesis and the accumulation of starch during amyloplast biogenesis in BY2 cells (Enami et al., 2011). *gun5* has less basal thermotolerance than wild type (Miller et al., 2007). Porphyrin signaling in general was suggested to promote oxidative stress tolerance (Zhang et al., 2011b). *gun5* and *cch* exhibit reduced cold tolerance relative to wild type (Kindgren et al., 2015). Overexpressing *Myxococcus xanthus* protoporphyrinogen IX oxidase in *Oryza sativa* enhanced drought tolerance relative to wild type (Phung et al., 2011). Although Phung et al. (2011) proposed that overexpressing protoporphyrinogen IX oxidase promoted drought tolerance by inducing increases in the levels of ROS that helped acclimate chloroplasts to stress, they did not detect elevated levels of ROS in plants that overexpressed protoporphyrinogen IX oxidase. Additionally, the lines overexpressing protoporphyrinogen IX oxidase exhibited an increased capacity for porphyrin biosynthesis that resulted in elevated levels of heme. Interesting, their data show a correlation between elevated levels of ferrochelatase activity, heme, and *Lhcb* expression, although the levels of heme appeared to increase only transiently (Phung et al., 2011). The data from Phung et al. (2011) are consistent with tetrapyrrole signaling, such as heme signaling, promoting drought tolerance. Indeed, there is a growing body of evidence supporting a role for heme signaling in drought tolerance in plants (Nagahatenna et al., 2015). Porphyrin-derived ROS, heme signaling, or both may contribute to drought tolerance in the transgenic plants described by Phung et al. (2011). A mutant allele that specifically disrupts tetrapyrrole-dependent plastid signaling without directly affecting an enzyme that contributes to tetrapyrrole biosynthesis would help to establish the biological function of tetrapyrrole-dependent plastid-to-nucleus signaling.

The interactions between GUN1- and tetrapyrrole-dependent plastid-to-nucleus signaling are consistent with additional biological functions for tetrapyrrole-dependent plastid-to-nucleus signaling because *GUN1* is known to promote diverse biological functions in plants. Loss-of-function alleles of *GUN1* affect chloroplast biogenesis (Mochizuki et al., 1996; Ruckle et al., 2007), plastid-to-nucleus signaling when plants are grown in diverse conditions (Susek et al., 1993; Koussevitzky et al., 2007; Ruckle et al., 2007; Kakizaki et al., 2009; Woodson et al., 2013; Tanaka et al., 2015; Tadini et al., 2016), the circadian rhythm (Hassidim et al., 2007), the accumulation of anthocyanins (Ruckle and Larkin, 2009; Cottage et al., 2010; Cheng et al., 2012), abiotic stress tolerance (Miller et al., 2007; Zhang et al., 2013), and the development of seedlings and leaves (Ruckle and Larkin, 2009; Cottage et al., 2010; Tameshige et al., 2013; Martín et al., 2016; Xu et al., 2016).

## Summary

Since the early days of plastid-to-nucleus signaling research, porphyrins were attractive candidates for plastid signals that regulate nuclear gene expression. A number of independent experiments with exogenous ALA, exogenous porphyrins, inhibitors, a variety of mutants with defects in tetrapyrrole metabolism, and both the overexpression and antisense suppression of enzymes that contribute to tetrapyrrole biosynthesis provide a large body of evidence favoring the idea that porphyrins regulate the expression of nuclear genes. Results from the *gun* mutant screen indicate that when chloroplast biogenesis is blocked, tetrapyrrole metabolism regulates the expression of PhANGs. There is a large body of evidence indicating that in the conditions used for the *gun* mutant screen, the effects of tetrapyrrole metabolism on nuclear gene expression are difficult or impossible to explain by changes in the levels of porphyrin-derived ROS. The regulation of nuclear gene expression by tetrapyrrole metabolism is conserved in *Arabidopsis*, barely, and maize. Thus, tetrapyrrole signaling makes an important contribution to plants.

**FIGURE 3:**
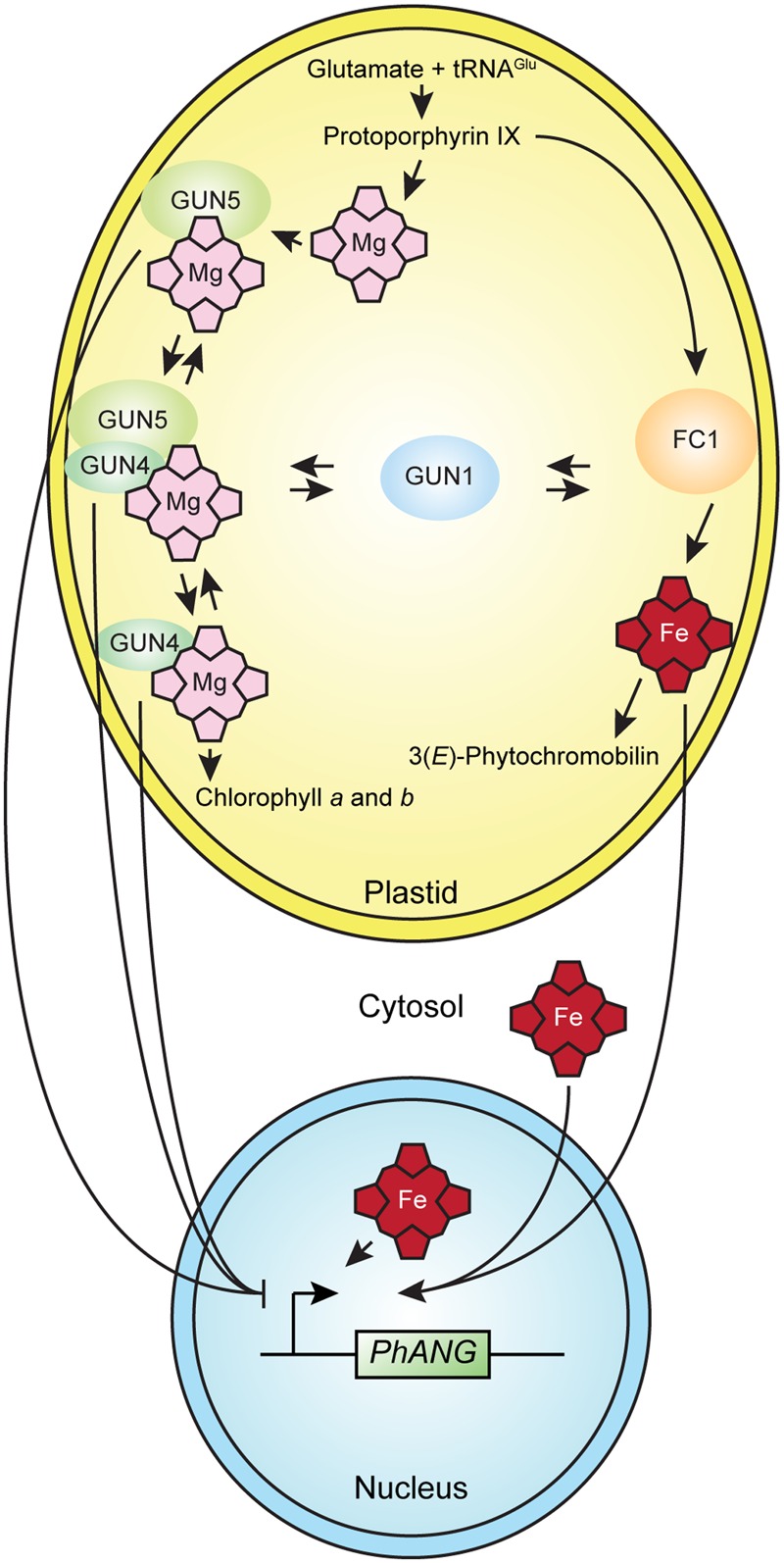
**A model for tetrapyrrole-dependent plastid-to-nucleus signaling in plants.** The simplest interpretation of the current data is that heme accumulates in well-functioning chloroplasts and that the accumulation of heme in chloroplasts or the export of heme to the cytosol or nucleus activates a mechanism that induces the expression of PhANGs. The current data do not exclude alternative mechanisms, such as a moonlighting function for an enzyme or regulatory factor that contributes to tetrapyrrole metabolism, such as GUN4, GUN5, or a GUN4-GUN5 complex that affects gene expression in the nucleus. Either the liganded or unliganded versions of GUN4, GUN5, or a GUN4-GUN5 complex might contribute to a plastid-to-nucleus signaling mechanism that regulates the expression of PhANGs. GUN1 interacts with tetrapyrrole-dependent plastid-to-nucleus signaling by an unknown mechanism.

The simplest interpretation of the current data is that in the conditions used for the *gun* mutant screen, heme serves as a plastid signal that regulates nuclear gene expression. The current data are also consistent with other mechanisms, such as a moonlighting function (Boukouris et al., 2016) for an enzyme or regulatory factor that contributes to tetrapyrrole metabolism in plastid-to-nucleus signaling, such as GUN5 (Mochizuki et al., 2001), GUN4, or a GUN4-GUN5 complex (Larkin et al., 2003) in plastid-to-nucleus signaling (**Figure 3**). The current data are also consistent with changes in the flux through the tetrapyrrole biosynthetic pathway activating a plastid-to-nucleus signaling mechanism (Mochizuki et al., 2008). There are precedents for each of these mechanisms in non-plant systems (Osanai et al., 2009; Terry and Smith, 2013). Interactions between tetrapyrrole-dependent plastid-to-nucleus signaling and GUN1-dependent plastid-to-nucleus signaling potentially link tetrapyrrole-dependent plastid-to-nucleus signaling to a network of plastidic and extraplastidic signaling mechanisms that is broadly significant.

The plastid-to-nucleus signaling mechanism that is specifically interrogated by the *gun* mutant screen in young seedlings does not appear to require a transient or sustained accumulation of Mg-Proto or Mg-ProtoME. However, in mature plants, the photooxidative stress that (1) induces increases in the levels of Mg-Proto and Mg-ProtoME and (2) is correlated with downregulated expression of PhANGs was observed under distinct developmental and environmental conditions relative to the conditions used to screen for *gun* mutants. The evidence consistent with oxidative stress inducing an increase in the levels of Mg-Proto or Mg-ProtoME that affects gene expression in the nucleus is also consistent with oxidative stress attenuating heme signaling or at least in some instances, ROS signaling

At this point, it is perhaps most important to understand the mechanism of tetrapyrrole signaling in plants. Understanding this mechanism may help us to understand why perturbations in tetrapyrrole metabolism sometimes upregulate, downregulate, or have no effect on the expression of nuclear genes. Understanding this mechanism may help us to understand the interactions between tetrapyrrole signaling and GUN1-dependent plastid-to-nucleus signaling, interactions between tetrapyrrole signaling and light-regulated development, and the biological function of tetrapyrrole signaling. Identifying a protein that specifically contributes to tetrapyrrole signaling (e.g., a protein that serves as a heme receptor in the plastid, cytosol, or nucleus) is perhaps the highest priority. Mutants with defects in such a gene are essential for learning the biological function of tetrapyrrole signaling in plants. The evidence for tetrapyrrole signaling contributing to abiotic stress tolerance and for the interactions between tetrapyrrole metabolism and GUN1-dependent plastid-to-to-nucleus signaling are consistent with an important role for tetrapyrrole signaling in plants. Thus, future research in this area is expected to advance our understanding of plant biology and possibly contribute to the development of new germplasm that will help to meet the needs of agriculture in our changing environment.

## Author Contributions

The author confirms being the sole contributor of this work and approved it for publication.

## Conflict of Interest Statement

The author declares that the research was conducted in the absence of any commercial or financial relationships that could be construed as a potential conflict of interest.
